# Risk Factors for Readmission Within 30 Days After Discharge Following Hip Fracture Surgery: A Systematic Review and Meta-Analysis

**DOI:** 10.3390/jcm14082779

**Published:** 2025-04-17

**Authors:** Kyung-Joo Lee, Ji Wan Kim, Chul-Ho Kim

**Affiliations:** 1 Department of Orthopaedic Surgery, National Police Hospital, Seoul 05715, Republic of Korea; lksjoo@naver.com; 2 Department of Orthopaedic Surgery, Asan Medical Center, University of Ulsan College of Medicine, Seoul 05505, Republic of Korea; jaykim@amc.seoul.kr

**Keywords:** hip fracture surgery, 30-day readmission, risk factors, comorbidities, meta-analysis

## Abstract

**Background/Objectives:** Hip fractures in older patients frequently lead to early readmissions, which negatively impact patient outcomes and significantly increase healthcare costs. Identifying and understanding risk factors for 30-day readmission following hip fracture surgery is essential for improving patient management and optimizing healthcare resource utilization. **Methods:** A systematic literature search was conducted using PubMed, EMBASE, and the Cochrane Library databases up to 30 December 2024. Studies investigating potential risk factors for 30-day readmission following hip fracture surgery were included. The risk factors were meta-analytically pooled, and odds ratios (ORs) were calculated using a random-effects model. **Results:** Twelve studies comprising 128,053 patients were included. Pooled analyses revealed significant associations between higher readmission rates and factors such as male sex (OR = 1.45; 95% CI, 1.27–1.65), hip arthroplasty surgery (OR = 1.36; 95% CI, 1.03–1.80), advanced age (OR, 1.22; 95% CI 1.00–1.49), high American Society of Anesthesiologists (ASA) Physical Status Classification System class (OR, 2.22; 95% CI, 1.28–3.85), and high Charlson comorbidity index (OR, 1.67; 95% CI, 1.36–2.05). Additionally, the most frequently reported comorbidities associated with higher readmission risks were diabetes mellitus and congestive heart failure, with ORs of 1.63 and 1.57, respectively. **Conclusions:** Male sex, advanced age, higher ASA scores, and greater preoperative comorbidity burdens significantly increase the risk of 30-day readmission following hip fracture surgery. Effective risk stratification and targeted perioperative management strategies addressing these identified factors may reduce early readmission rates and enhance postoperative patient outcomes.

## 1. Introduction

Hip fractures represent a major public health challenge among older adults, often resulting in significant morbidity, mortality, and substantial economic burden [1]. With global populations aging, the incidence of hip fractures continues to rise, and these injuries are frequently compounded by multiple comorbidities and frailty. Despite advancements in surgical techniques and perioperative care, early hospital readmissions remain alarmingly common. These readmissions serve as critical indicators of healthcare quality and patient outcomes. According to a study by Jencks et al., the cost to Medicare for unplanned readmissions in a single year was estimated to exceed USD 17 billion [2]. Similarly, the rate of early readmissions after hip fractures continues to rise, with a 41.2% increase in 28-day readmissions in the United Kingdom over the past 12 years [3].

Unplanned readmissions following hip fractures have been linked to adverse clinical outcomes, including increased mortality, impaired functional recovery, and escalated healthcare costs [4]. In light of these challenges, understanding the underlying risk factors for early readmission is essential for guiding improvements in patient management and healthcare resource allocation.

Previous studies have explored various predictors for 30-day readmission after hip fracture surgery. These predictors include patient demographics, preoperative health status, and perioperative management strategies [5,6]. Notably, factors, such as age, sex, comorbidities (e.g., diabetes, congestive heart failure), surgical technique, and postoperative complications, have frequently been examined in prior studies. However, their relative impact on readmission rates remains controversial. Although some studies report a strong association between specific factors—such as high American Society of Anesthesiologists (ASA) Physical Status Classification System scores or the presence of cardiovascular diseases—and early readmission, others have failed to replicate these findings. Variability in reported risk factors may result from differences in study design and sample size. Other contributing factors include varying healthcare system characteristics and differing definitions of readmission. Furthermore, regional variations in discharge planning, rehabilitation availability, and follow-up care may also contribute to these discrepancies.

Therefore, the objective of this systematic review and meta-analysis was to identify and quantitatively synthesize the significant risk factors associated with 30-day readmission following hip fracture surgery.

## 2. Materials and Methods

We conducted this study in accordance with the Revised Assessment of Multiple Systematic Reviews and the Preferred Reporting Items for Systematic Reviews and Meta-Analyses guidelines [7,8]. Although this research involved human participants, ethical approval was not required for this systematic review and meta-analysis as all analyses were based on previously published, publicly available data, and no new patient data or interventions were involved. This study adheres to the principles outlined in the Declaration of Helsinki.

### 2.1. Literature Search

A computerized search of MEDLINE (PubMed), Embase, and the Cochrane Library was conducted to identify studies analyzing risk factors for short-term readmission after hip fracture surgery. Using a predefined search strategy, we identified articles published up to 30 December 2024. The search terms included synonyms and related terms for hip fracture and readmission. The full search strategies and results for all databases are presented in Supplementary Materials S1. No language restrictions were applied to our literature search, and we placed no restrictions on the publication year. Following the initial electronic search, we manually screened relevant articles and their bibliographies.

### 2.2. Study Selection

Two board-certified orthopedic surgeons (K.-J.L. and C.-H.K.) independently selected the articles for full-text review based on the titles and abstracts of the studies. If the abstract provided insufficient data to decide, the entire article was reviewed. Paper inclusion was based on the Patient/Intervention/Comparator/Outcome/Study design (PICOS) criteria [9]: (1) “Patient” was set as patients who underwent hip fracture; (2) “Intervention” was set as hip fracture surgery; (3) 30-day readmission was set as the “Comparator”; and (4) “Outcomes” were investigated for all variables.

**Figure 1:**
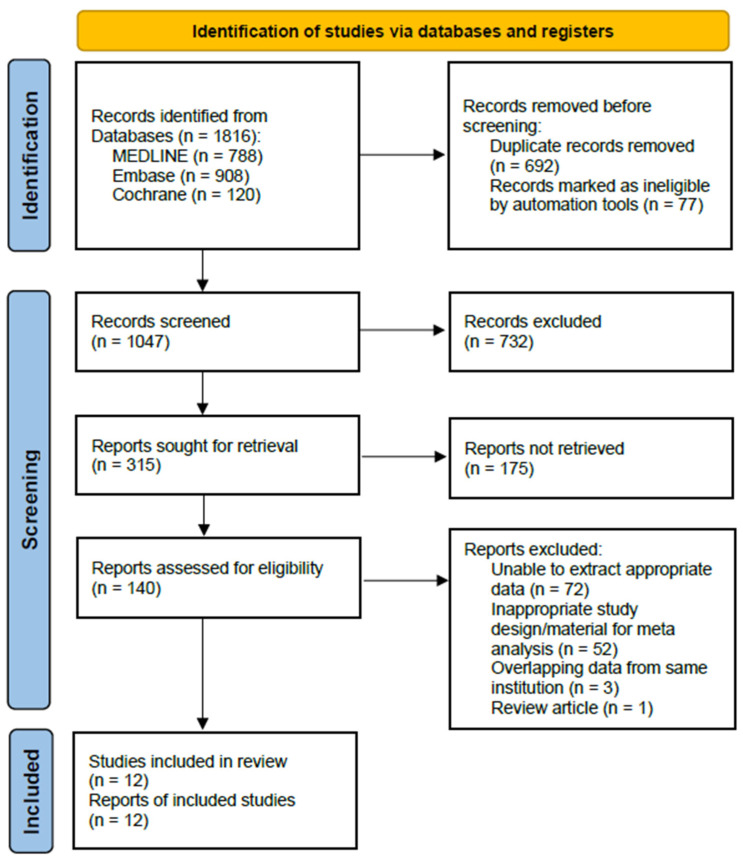
Preferred Reporting Items for Systematic Reviews and Meta-Analyses (PRISMA) 2020 flow diagram for identifying and selecting the studies included in this meta-analysis.

This systematic review and meta-analysis were performed in accordance with the Preferred Reporting Items for Systematic Reviews and Meta-Analyses (PRISMA) guidelines. We included only original articles, and the exclusion criteria were as follows: papers from which appropriate data could not be extracted despite contacting the author, and cases where study populations overlapped, in which we selected the publication with the largest population for the meta-analysis. The process of identifying and selecting studies is outlined in Figure 1.

At each stage of article selection, the κ-value was calculated to determine inter-reviewer agreement regarding study selection. Agreement between reviewers was correlated a priori with κ-values as follows: κ = 1 corresponded to “perfect” agreement, 1.0 > κ ≥ 0.8 to “almost perfect” agreement, 0.8 > κ ≥ 0.6 to “substantial” agreement, 0.6 > κ ≥ 0.4 to “moderate” agreement, 0.4 > κ ≥ 0.2 to “fair” agreement, and κ < 0.2 to “slight” agreement. Disagreements at each stage were resolved by discussion between the two investigators to reach a consensus or by a discussion with a third investigator (J.W.K), who was a board-certified orthopedic faculty member, when a consensus could not be reached.

### 2.3. Data Extraction

For qualitative data synthesis, the following information and variables were extracted using a standardized form: (1) study design, (2) the country in which the investigation took place, (3) mean patient age, (4) sex, (5) type of hip surgery, (6) overall readmission rate, and (7) potential risk factors investigated.

The potential risk factors examined in each included study were ranked based on frequency, and the top five most frequently investigated variables were identified. Among all included papers, the top five risk factors were sex, type of surgery, older age, high ASA score, and high Charlson comorbidity index (CCI). A meta-analysis was conducted using these five key risk factors.

Additionally, we did not include underlying comorbidities in the top five most frequent potential risk factors mentioned earlier. This decision was made because the classification of comorbidities varied across individual studies and was highly heterogeneous. Instead, comorbidities were categorized separately, and a meta-analysis was conducted on the most frequently reported underlying comorbidities: diabetes mellitus (DM) and congestive heart failure (CHF).

### 2.4. Statistical Analysis

A meta-analysis was conducted to analyze the risk factors for 30-day readmission after hip fracture. Univariate odds ratios (ORs) and their 95% confidence intervals (CIs) were calculated. We pooled all data using a random-effects model, as recommended in the medical field, to avoid overestimating the study results [10]. The fixed-effects model begins with the assumption that the true effect size is similar in all included studies; thus, we hypothesized that the random-effects model was generally a more plausible match for the current study. For analysis metrics with more than 10 included studies, publication bias was assessed following Cochrane’s recommendations [10]. All statistical analyses were performed using the “metafor” package in R (R Foundation for Statistical Computing, version 4.3.0).

## 3. Results

### 3.1. Article Identification

Details of the study identification and selection processes are summarized in Figure 1. The initial electronic literature search yielded 1816 articles. After removing 769 duplicates and ineligible articles using automated tools, 1047 articles remained and were screened. Of these, we excluded 732 articles after checking the title of reports, and then, 175 articles were excluded after reviewing the abstracts. Finally, after examining the content of the text, 128 articles were excluded because appropriate data for analysis could not be extracted, they had overlapping research subjects, or they were review articles. Thus, 12 studies were eligible for qualitative and quantitative data syntheses in the current meta-analysis. The κ-values between the two reviewers were 0.756 at the title review stage and 0.782 at the abstract review stage, indicating substantial agreement; this value was 1.000 at the full-text review stage, indicating perfect agreement.

### 3.2. Study Characteristics and Qualitative Synthesis

Of the 12 studies [5,6,11,12,13,14,15,16,17,18,19,20], except for one randomized controlled trial [16], all the others had a retrospective study design. The majority of the studies (8 out of 12) were conducted in North America [6,11,12,13,14,17,18,19], followed by 3 studies from Europe [15,16,20] and 1 from Asia [5].

The mean age of the study participants ranged from 74.9 to 85.1 years, and in all studies, the proportion of female patients was higher. Information on the type of surgery was available for all but two studies [13,14], with most procedures being either hip arthroplasty or osteosynthesis. The average 30-day readmission rate ranged from 8.1% to 15.9%. Additional details on the investigated potential risk factors for readmission are presented in Table 1.

### 3.3. Risk-of-Bias Assessment

The methodological quality and risk of bias of the included studies were assessed using the MINORS tool [21] (Table 1). The mean MINORS score for methodological quality assessment was 18.58/24, and the scores ranged from 18 to 21 out of a possible 24 points, indicating moderate to high overall methodological quality among the selected studies. The most common methodological limitations identified included retrospective study designs, lack of prospective sample size calculations, and baseline differences in patient characteristics between readmitted and non-readmitted groups. Despite these limitations, the majority of studies exhibited clearly defined aims, comprehensive inclusion of consecutive patients, appropriate and relevant study endpoints, adequate follow-up periods, and robust statistical analyses. Therefore, the evidence synthesized from these studies was considered sufficiently reliable for the purposes of this meta-analysis.

### 3.4. Meta-Analysis

#### 3.4.1. Top Five Reasons for 30-Day Readmission Following Hip Surgery

Among the 12 papers included [5,6,11,12,13,14,15,16,17,18,19,20], past medical history was excluded due to inconsistent analysis. The top five reasons were determined based on the frequency with which the remaining indicators were analyzed. The most frequently identified risk factor was sexual difference (12 out of 12 papers), followed by the type of surgery—whether hip arthroplasty or osteosynthesis (8 out of 12 papers). Next, both older age and high ASA scores were analyzed in 6 out of the 12 papers, making them equally common, and high CCI score was investigated in 2 papers, ranking it as the fifth most studied factor.

**Figure 2:**
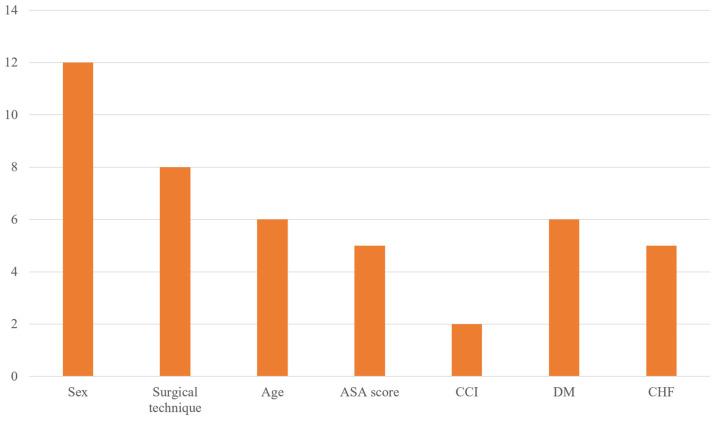
Most frequently investigated potential risk factors for 30-day readmission after hip fracture surgery in included studies.

For older age, the majority of papers (four out of six) calculated the OR by dividing participants into older age and control groups using 90 years as the cutoff, whereas the remaining two papers used 85 and 80 years, respectively. In all six papers, the ASA score was used to split subjects into groups based on a cutoff of ≥3 points, and the CCI score was similarly grouped using a cutoff of ≥4 points. Additional details are shown as a bar graph in Figure 2.

This figure demonstrates the odds ratios for significant demographic and perioperative predictors of emergency room visits using multivariate logistic regression analysis.

#### 3.4.2. Sexual Differences

**Figure 3:**
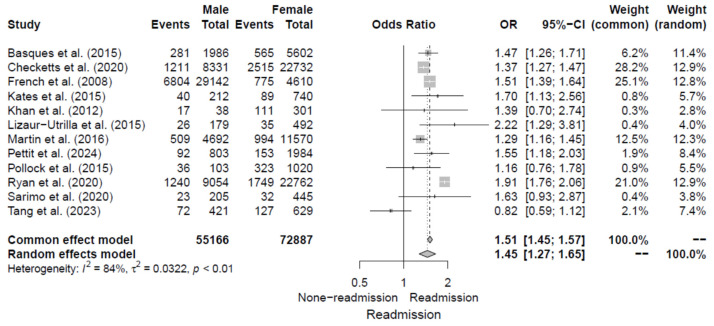
Forest plot of odds ratios for male sex in relation to 30-da readmission risk following hip fracture [5,6,11,12,13,14,15,16,17,18,19,20].

**Figure 4:**
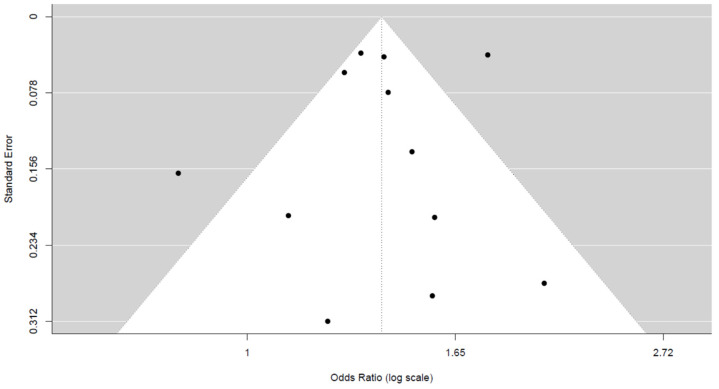
Funnel plots and Egger’s test show that no significant publication bias was present in the studies assessing sexual differences.

All of the 12 included studies [5,6,11,12,13,14,15,16,17,18,19,20] evaluated sexual differences as a risk factor for 30-day readmission following hip fracture by reporting ORs. The pooled OR for the male sex was 1.45 (95% CI, 1.27–1.65), with significant heterogeneity of I^2^ = 84%. Additional details are shown in Figure 3. Also, no publication bias was observed upon visual assessment of the funnel plot (Figure 4) and Egger’s test (*p* = 0.792).

#### 3.4.3. Type of Surgery

**Figure 5:**
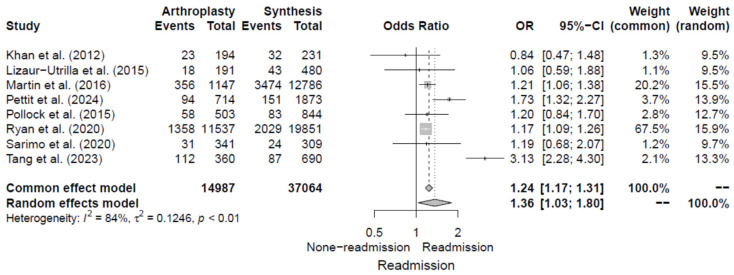
Forest plot of odds ratios for type of surgery in relation to 30-day readmission risk following hip fracture [5,6,15,16,17,18,19,20].

Eight studies [5,6,15,16,17,18,19,20] analyzed whether the type of surgery was associated with the risk of 30-day readmission following hip fracture surgery and presented the corresponding ORs. The pooled OR for hip arthroplasty was 1.36 (95% CI, 1.03–1.80) compared to osteosynthesis, with significant heterogeneity of I^2^ = 84%. Additional details are shown in Figure 5.

#### 3.4.4. Older Age

**Figure 6:**
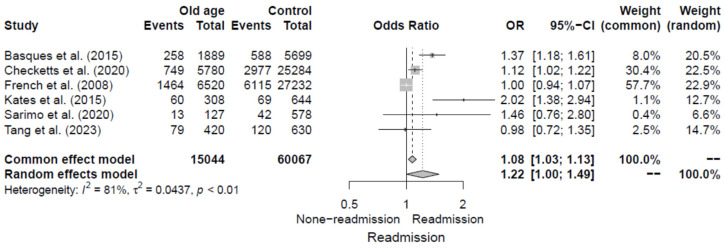
Forest plot of odds ratios for older age in relation to 30-day readmission risk following hip fracture [5,11,12,13,14,20].

The six studies [5,11,12,13,14,20] evaluated older age as a risk factor for 30-day readmission following hip fracture by reporting ORs. Variations were observed in the definition of older age across the studies when categorizing the study population into binary groups. Among the six studies, four [11,12,14,20] set 90 years as the cut-off value, one [13] set 85 years, and one [5] set 80 years. The pooled OR for older age was 1.22 (95% CI, 1.00–1.49), with significant heterogeneity of I^2^ = 81%. Additional details are shown in Figure 6.

#### 3.4.5. High ASA Score

**Figure 7:**
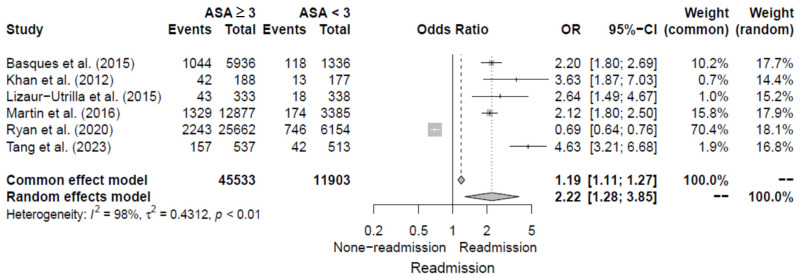
Forest plot of odds ratios for high ASA score in relation to 30-day readmission risk following hip fracture [5,11,15,16,17,19].

The six studies [5,11,15,16,17,19] evaluated high ASA score (≥3 points) as a risk factor for 30-day readmission following hip fracture by reporting ORs. The pooled OR for high ASA scores was 2.22 (95% CI, 1.28–3.85), with significant heterogeneity of I^2^ = 98%. Additional details are shown in Figure 7.

#### 3.4.6. High CCI Score

**Figure 8:**
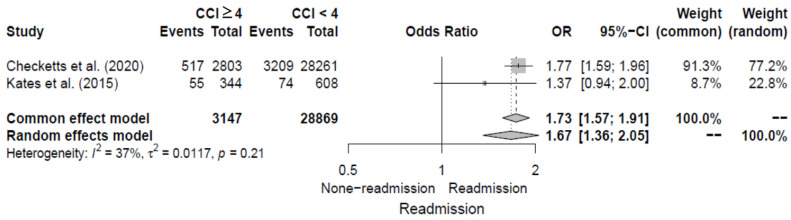
Forest plot of odds ratios for high CCI score in relation to 30-day readmission risk following hip fracture [12,14].

Two studies [12,14] evaluated high CCI score (≥4 points) as a risk factor for 30-day readmission following hip fracture by reporting ORs. The pooled OR for high CCI scores was 1.67 (95% CI, 1.36–2.05), with moderate heterogeneity of I^2^ = 37%. Additional details are shown in Figure 8.

#### 3.4.7. Underlying Comorbidity: DM and CHF

Six studies analyzed DM as an underlying comorbidity and risk factor for 30-day readmission following hip fracture, whereas five studies analyzed CHF.

**Figure 9:**
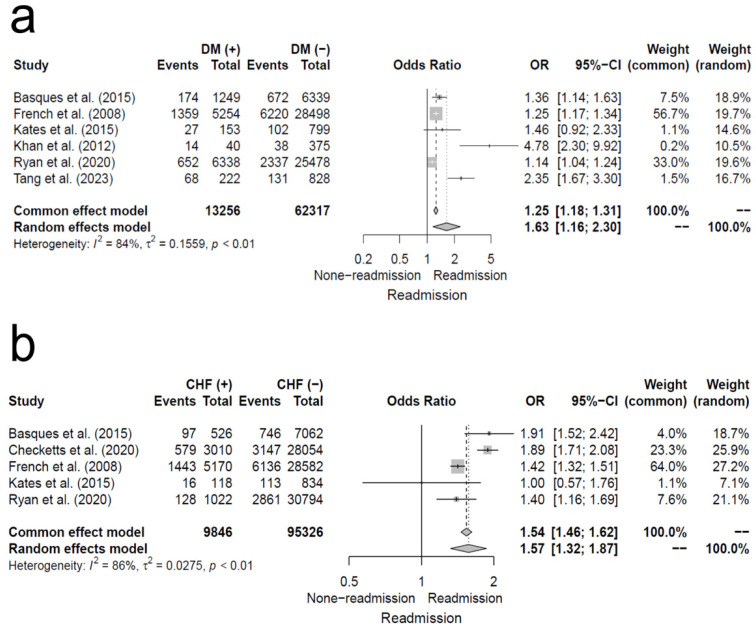
Forest plot of odds ratios for (**a**) underlying patient with diabetes mellitus, and (**b**) underlying patient with congestive heart failure in relation to 30-day readmission risk following hip fracture [5,11,12,13,14,15,19].

The pooled OR for patients with underlying DM, compared to the control group, was 1.63 (95% CI, 1.16–2.30; I^2^ = 84%), indicating a significantly higher 30-day readmission rate. Similarly, for patients with underlying CHF, the pooled OR was 1.57 (95% CI, 1.32–1.87; I^2^ = 86%), showing a significantly increased risk of 30-day readmission compared to the control group. Additional details are provided in Figure 9.

## 4. Discussion

The findings of the current meta-analysis indicate that, among the top five risk factors for readmission within 30 days following a hip fracture, (1) males have a 1.45 times higher readmission rate than females; (2) undergoing hip arthroplasty is associated with a 1.36 times higher readmission rate compared to osteosynthesis; (3) advanced age is associated with a 1.22 times higher readmission rate compared to the control group; (4) a preoperative ASA score of 3 or higher is associated with a 2.22 times higher readmission rate; and (5) a preoperative CCI score of 4 or higher is associated with a 1.67 times higher readmission rate. Regarding underlying comorbidities, patients with DM had a 1.63 times higher likelihood of readmission compared to the control group, whereas patients with CHF had a 1.57 times higher likelihood of readmission.

It is well established in the field of orthopedic hip surgery that males have higher mortality and morbidity rates compared to females. According to a study conducted by Kim et al. on an Asian population [22], the one-year mortality rate after a hip fracture was 6% higher in males than in females. Additionally, a European study by Olalla et al. [23], utilizing national registry data, confirmed that male sex remained a significant risk factor for increased mortality following hip fracture surgery, even after adjusting for other variables. Given these previous findings, male sex is also, unsurprisingly, a risk factor for short-term readmission following hip surgery; this is likely because despite their smaller numbers, male patients tend to have a higher preoperative comorbidity burden compared to female patients.

Secondly, the type of surgery also influenced the likelihood of readmission, with hip arthroplasty being associated with a 1.36 times higher 30-day readmission rate than the control group. We speculate that this is due to the increased short-term risks of complications, such as dislocation and infection, following hip arthroplasty compared to other procedures. In fact, previous literature has identified prosthesis dislocation and postoperative infection following arthroplasty as the most common causes of short-term readmission after hip surgery [24,25]. Furthermore, a retrospective analysis of 4551 patients with hip fractures at our institution revealed that among patients readmitted within 30 days due to surgical complications excluding medical complications, dislocation following hip arthroplasty surgery was the most common surgical cause, occurring in 9.2% of cases in overall 30-day readmission. Therefore, greater attention to surgical technique is warranted when performing arthroplasty to minimize these complications.

Next, older age was identified as a significant factor increasing the likelihood of short-term readmission (pooled OR, 1.22). Although it was not included in this meta-analysis, as it did not meet our inclusion criteria, a large-cohort study analyzing 514,455 patients using UK National Health Service data previously reported that increasing age was strongly associated with 30-day readmission following hip arthroplasty [26]. Furthermore, a recent study by Yohe et al. [27] found that when total hip arthroplasty was performed in patients aged 80 years and older, the unplanned readmission rate was significantly higher compared to younger patients (OR, 2.4). Considering these findings, our study results are in line with previous research and can be regarded as reasonable and consistent outcomes from previous studies.

Although the findings are not limited to the field of orthopedic hip surgery, one study reported that the pre-injury ASA score is a predictor of 30-day readmission following major traumatic injury [28]. Moreover, this study suggested that the ASA score is an even better predictor of readmission compared to the CCI. Similarly, in the current study, although both a high preoperative ASA score and a high preoperative CCI were identified as significant risk factors for 30-day readmission, the pooled OR for an ASA score ≥ 3 was 2.22, whereas the pooled OR for a high CCI was 1.67, indicating that a high ASA score is a stronger predictor of short-term readmission following hip surgery.

The analysis of comorbidities was highly heterogeneous, making systematic evaluation challenging. Among the most frequently investigated comorbidities, DM and CHF were the most commonly studied, as data extraction was possible from at least three studies. These two conditions are well known as poor prognostic factors in orthopedic surgeries for older patients [29,30].

Ultimately, identifying both modifiable and non-modifiable predictors will help guide targeted interventions aimed at reducing readmission rates and improving overall patient outcomes in this population. Comorbidities, sex, age, and preoperative physical status scores (ASA or CCI) are non-modifiable risk factors. However, surgical techniques and procedural precision represent modifiable aspects that clinicians can optimize. Therefore, greater attention should be given to these controllable factors to minimize the risk of readmission.

This study had some limitations. First, due to its meta-analytical nature, the findings were inevitably influenced by the included studies. Most of these studies had retrospective designs, potentially limiting the generalizability of our results. Nonetheless, this meta-analysis is the first to systematically examine risk factors associated with 30-day readmission after hip fracture surgery, analyzing the largest available cohort, which underscores its clinical significance. Second, as mentioned earlier, preoperative comorbidity is a major factor contributing to readmission during the acute postoperative stage. However, high heterogeneity (I² > 80%) likely arises from variability in study populations, healthcare practices, differing definitions of readmission, and variations in surgical techniques across the included studies. Furthermore, potential limitations such as publication bias, variability in data standardization, and methodological heterogeneity among the included studies should be considered when interpreting our findings. These issues warrant careful consideration and should be addressed in future research.

## 5. Conclusions

Among all the included papers, the top five risk factors for 30-day readmission after hip surgery were male sex, hip arthroplasty surgery, older age, high ASA score, and high CCI. The most frequently investigated comorbidities identified as potential risk factors were DM and CHF.

## Figures and Tables

**Table 1:** Information from 12 articles included for meta-analysis.

Author (Year)	Study Design	Country	Mean Age	Sex (Male/Female, %)	Type of Hip Surgery	Overall Readmission Rate (%)	Investigated Potential Risk Factors	Minors Score
Basques et al. (2015) [11]	RCS (ACS-NSQIP data)	USA	83.8 ± 5.9	26.9/73.1	Percutaneous fixation, HA, THA, plate/screw, IM implant	10.0	Age, sex, ASA class, BMI, functional status, discharge location, comorbidities	19
Checketts et al. (2020) [12]	RCS (CHFEHR data)	USA	81.3 ± 6.4	27.4/72.6	Hip replacement, other methods	10.7	Race, sex, age, discharge location, CCI, length of stay (LOS), blood transfusion	18
French et al. (2008) [13]	RCS (VIReC data)	USA	79.9 ± 6.3	87.0/13.0	N/A	18.3	Age, sex, LOS, comorbidities	18
Kates et al. (2015) [14]	RCS	USA	85.1 ± 8.4	24.0/76.0	N/A	11.9	Age, sex, Parker mobility score, CCI, comorbidities, in-hospital complication	18
Khan et al. (2012) [15]	RCS	UK	79.6	11.8/88.2	Cannulated hip screws, DHS, cemented HA, uncemented HA, IM fixation	11.8	Age, sex, fracture type, functional status, ASA class, LOS, discharge location, comorbidities	19
Lizaur-Utrilla et al. (2015) [16]	RCT	Spain	82.0	28.1/71.9	Internal fixation, arthroplasty	8.3	Age, sex, ASA class, CCI, LOS, discharge location, comorbidities	20
Martin et al. (2016) [17]	RCS (ACS-NSQIP data)	USA	79.9	29.3/70.7	HA, THA, ORIF	8.5	Age, sex, ASA class, BMI, LOS, comorbidities	21
Pettit et al. (2024) [18]	RCS	USA	82.1	29.5/70.5	HA, THA, CRPP, IM nail, DHS	8.1	Age, sex, race, BMI, CCI, ASA class, LOS	18
Pollock et al. (2015) [6]	RCS	USA	82.0	24.4/75.6	Replacement, fixation	9.3	Age, Sex, ISS score, discharge location, LOS, comorbidities	18
Ryan et al. (2020) [19]	RCS (ACS-NSQIP data)	Canada	79.2 ± 7.8	29.6/70.4	IM nail, arthroplasty, ORIF	8.6	Age, sex, BMI, race, ASA class, functional status, anesthesia type, low hematocrit, transfusion	18
Sarimo et al. (2020) [20]	RCS	Finland	85	32.9/67.1	HA, IM nail, plate/screw fixation	8.3	Age, sex, fracture type, LOS, day of surgery/discharge	18
Tang et al.(2020) [5]	RCS	China	74.9	39.1/60.9	THA, HA, IM nail, plate/screw fixation, screw fixation	15.9	Age, sex, ASA class, type of fracture, laboratory findings, comorbidities	18

RCS: retrospective cohort study; RCT: randomized controlled trial; N/A: not available; HA: hemi-arthroplasty; THA: total hip arthroplasty; IM: intra-medullary; ASA: American Society of Anesthesiologists; BMI: body mass index; CCI: Charlson comorbidity index; LOS: length of stay; ISS: injury severity score; MINORS: methodological index for non-randomized studies.

## Data Availability

The original contributions presented in this study are included in the article/Supplementary Material. Further inquiries can be directed to the corresponding author.

## Supplementary Materials

The following supporting information can be downloaded at: https://www.mdpi.com/article/10.3390/jcm14082779/s1, Search term related materials S1.

## Abbreviations

ORs	odds ratios
ASA	American Society of Anesthesiologists
PICOS	Patient/Intervention/Comparator/Outcome/Study design
CCI	Charlson comorbidity index
DM	diabetes mellitus
CHF	congestive heart failure
MINORS	methodological index for non-randomized studies
CIs	confidence intervals
RCS	retrospective cohort study
RCT	randomized controlled trial
N/A	not available
HA	hemi-arthroplasty
THA	total hip arthroplasty
IM	intra-medullary
BMI	body mass index
LOS	length of stay
ISS	injury severity score

## References

[1] Sing C.W., Lin T.C., Bartholomew S., Bell J.S., Bennett C., Beyene K., Bosco-Levy P., Bradbury B.D., Chan A.H.Y., Chandran M. (2023). Global Epidemiology of Hip Fractures: Secular Trends in Incidence Rate, Post-Fracture Treatment, and All-Cause Mortality. J. Bone Miner. Res..

[2] Jencks S.F., Williams M.V., Coleman E.A. (2009). Rehospitalizations among Patients in the Medicare Fee-for-Service Program. N. Engl. J. Med..

[3] Ali A.M., Gibbons C.E.R. (2017). Predictors of 30-day hospital readmission after hip fracture: A systematic review. Injury.

[4] Chang J.-K., Huang H.-T., Ho M.-L., Lin H.-T., Ho P.-S., Lee T.-C. (2017). One-Year Readmission Risk and Mortality after Hip Fracture Surgery: A National Population-Based Study in Taiwan. Aging Dis..

[5] Tang W.Y., Yao W., Wang W., Lv Q.M., Ding W.B., He R.J. (2023). Development and validation of a nomogram for 30-day readmission after hip fracture surgery in geriatric patients. Eur. Rev. Med. Pharmacol. Sci..

[6] Pollock F.H., Bethea A., Samanta D., Modak A., Maurer J.P., Chumbe J.T. (2015). Readmission Within 30 Days of Discharge After Hip Fracture Care. Orthopedics.

[7] Moher D., Shamseer L., Clarke M., Ghersi D., Liberati A., Petticrew M., Shekelle P., Stewart L.A., Group P.-P. (2015). Preferred reporting items for systematic review and meta-analysis protocols (PRISMA-P) 2015 statement. Syst. Rev..

[8] Shea B.J., Grimshaw J.M., Wells G.A., Boers M., Andersson N., Hamel C., Porter A.C., Tugwell P., Moher D., Bouter L.M. (2007). Development of AMSTAR: A measurement tool to assess the methodological quality of systematic reviews. BMC Med. Res. Methodol..

[9] Moher D., Liberati A., Tetzlaff J., Altman D.G., Group P. (2009). Preferred reporting items for systematic reviews and meta-analyses: The PRISMA statement. PLoS Med..

[10] Schmidt F.L., Oh I.S., Hayes T.L. (2009). Fixed- versus random-effects models in meta-analysis: Model properties and an empirical comparison of differences in results. Br. J. Math. Stat. Psychol..

[11] Basques B.A., Bohl D.D., Golinvaux N.S., Leslie M.P., Baumgaertner M.R., Grauer J.N. (2015). Postoperative length of stay and 30-day readmission after geriatric hip fracture: An analysis of 8434 patients. J. Orthop. Trauma.

[12] Checketts J.X., Dai Q., Zhu L., Miao Z., Shepherd S., Norris B.L. (2020). Readmission Rates After Hip Fracture: Are There Prefracture Warning Signs for Patients Most at Risk of Readmission?. J. Am. Acad. Orthop. Surg..

[13] French D.D., Bass E., Bradham D.D., Campbell R.R., Rubenstein A.L.Z. (2007). Rehospitalization After Hip Fracture: Predictors and Prognosis from a National Veterans Study. J. Am. Geriatr. Soc..

[14] Kates S.L., Behrend C., Mendelson D.A., Cram P., Friedman S.M. (2014). Hospital readmission after hip fracture. Arch. Orthop. Trauma Surg..

[15] Khan M.A., Hossain F.S., Dashti Z., Muthukumar N. (2012). Causes and predictors of early re-admission after surgery for a fracture of the hip. J. Bone Jt. Surg. Br..

[16] Lizaur-Utrilla A., Serna-Berna R., Lopez-Prats F.A., Gil-Guillen V. (2015). Early rehospitalization after hip fracture in elderly patients: Risk factors and prognosis. Arch. Orthop. Trauma Surg..

[17] Martin C.T., Gao Y., Pugely A.J. (2016). Incidence and Risk Factors for 30-Day Readmissions After Hip Fracture Surgery. Iowa Orthop. J..

[18] Pettit C.J., Herbosa C.F., Ganta A., Rivero S., Tejwani N., Leucht P., Konda S.R., Egol K.A. (2024). Can We Predict 30-day Readmission Following Hip Fracture?. J. Orthop. Trauma.

[19] Ryan G., Nowak L., Melo L., Ward S., Atrey A., Schemitsch E.H., Nauth A., Khoshbin A. (2020). Anemia at Presentation Predicts Acute Mortality and Need for Readmission Following Geriatric Hip Fracture. JBJS Open Access.

[20] Sarimo S., Pajulammi H., Jämsen E. (2020). Process-related predictors of readmissions and mortality following hip fracture surgery: A population-based analysis. Eur. Geriatr. Med..

[21] Slim K., Nini E., Forestier D., Kwiatkowski F., Panis Y., Chipponi J. (2003). Methodological index for non-randomized studies (minors): Development and validation of a new instrument. ANZ J. Surg..

[22] Kim B.-S., Lim J.-Y., Ha Y.-C. (2020). Recent Epidemiology of Hip Fractures in South Korea. Hip Pelvis.

[23] Guzon-Illescas O., Perez Fernandez E., Crespí Villarias N., Quirós Donate F.J., Peña M., Alonso-Blas C., García-Vadillo A., Mazzucchelli R. (2019). Mortality after osteoporotic hip fracture: Incidence, trends, and associated factors. J. Orthop. Surg. Res..

[24] Phruetthiphat O.-a., Otero J.E., Zampogna B., Vasta S., Gao Y., Callaghan J.J. (2020). Predictors for readmission following primary total hip and total knee arthroplasty. J. Orthop. Surg..

[25] Wang T., Gao C., Wu D., Li C., Cheng X., Yang Z., Zhang Y., Zhu Y. (2023). One-year unplanned readmission after total hip arthroplasty in patients with osteonecrosis of the femoral head: Rate, causes, and risk factors. BMC Musculoskelet. Disord..

[26] Ali A.M., Loeffler M.D., Aylin P., Bottle A. (2017). Factors Associated with 30-Day Readmission after Primary Total Hip Arthroplasty: Analysis of 514 455 Procedures in the UK National Health Service. JAMA Surg..

[27] Yohe N., Weisberg M.D., Ciminero M., Mannino A., Erez O., Saleh A. (2020). Complications and Readmissions After Total Hip Replacement in Octogenarians and Nonagenarians. Geriatr. Orthop. Surg. Rehabil..

[28] Tran A., Mai T., El-Haddad J., Lampron J., Yelle J.-D., Pagliarello G., Matar M. (2017). Preinjury ASA score as an independent predictor of readmission after major traumatic injury. Trauma Surg. Acute Care Open.

[29] Cantrell C.K., DeBell H.A., Lehtonen E.J., Patel H.A., McKissack H.M., McGwin G., Shah A., Naranje S. (2020). Risk factors for readmission within thirty days following revision total hip arthroplasty. J. Clin. Orthop. Trauma.

[30] Higuera C.A., Elsharkawy K., Klika A.K., Brocone M., Barsoum W.K. (2011). 2010 Mid-America Orthopaedic Association Physician in Training Award: Predictors of Early Adverse Outcomes after Knee and Hip Arthroplasty in Geriatric Patients. Clin. Orthop. Relat. Res..

