# Supplementary material for: Risk Factors for Readmission Within 30 Days After Discharge Following Hip Fracture Surgery: A Systematic Review and Meta-Analysis
Source: J Clin Med. 2025 Apr 17;14(8):2779. doi: 10.3390/jcm14082779 (PMC12028098; doi:10.3390/jcm14082779)
Supplement: Supplementary file 1 [file jcm-14-02779-s001.zip › jcm-3568110-supplementary.pdf]

Supplementary Materials S1. Literature search algorithm and results from relevant clinical studies

PubMed (January 31, 2025)

	Search Queries	Articles #
#1	hip fracture* [Title/Abstract]	23,068
#2	hip fractures [MeSH Terms]	30,803
#3	1 OR 2	38,735
#4	readmission* [Title/Abstract]	50,350
#5	patient readmission [MeSH Terms]	24,791
#6	1 AND 5	57,047
#7	3 AND 6	788

Embase (January 31, 2025)

	Search Queries	Articles #
#1	hip fracture*:ti,ab,kw	32,932
#2	readmission*:ti,ab,kw	91,690
#3	1 AND 2	908

Cochrane Library (January 31, 2025)

	Search Queries	Articles #
#1	hip fracture*:ti,ab,kw	7,846
#2	Readmission*: MeSH Terms	9,545
#6	1 AND 2	120
